# Modeling Calcium Cycling in the Heart: Progress, Pitfalls, and Challenges

**DOI:** 10.3390/biom12111686

**Published:** 2022-11-14

**Authors:** Zhilin Qu, Dasen Yan, Zhen Song

**Affiliations:** 1 Department of Medicine, David Geffen School of Medicine, University of California, A2-237 CHS, 650 Charles E. Young Drive South, Los Angeles, CA 90095, USA; 2 Department of Computational Medicine, David Geffen School of Medicine, University of California, Los Angeles, CA 90095, USA; 3 Peng Cheng Laboratory, Shenzhen 518066, China

**Keywords:** calcium cycling, excitation–contraction coupling, arrhythmias, computer modeling

## Abstract

Intracellular calcium (Ca) cycling in the heart plays key roles in excitation–contraction coupling and arrhythmogenesis. In cardiac myocytes, the Ca release channels, i.e., the ryanodine receptors (RyRs), are clustered in the sarcoplasmic reticulum membrane, forming Ca release units (CRUs). The RyRs in a CRU act collectively to give rise to discrete Ca release events, called Ca sparks. A cell contains hundreds to thousands of CRUs, diffusively coupled via Ca to form a CRU network. A rich spectrum of spatiotemporal Ca dynamics is observed in cardiac myocytes, including Ca sparks, spark clusters, mini-waves, persistent whole-cell waves, and oscillations. Models of different temporal and spatial scales have been developed to investigate these dynamics. Due to the complexities of the CRU network and the spatiotemporal Ca dynamics, it is challenging to model the Ca cycling dynamics in the cardiac system, particularly at the tissue sales. In this article, we review the progress of modeling of Ca cycling in cardiac systems from single RyRs to the tissue scale, the pros and cons of the current models and different modeling approaches, and the challenges to be tackled in the future.

## 1. Introduction

The heart is probably the most intensively and accurately modeled biological system compared to other organs [1,2,3,4,5]. So far, more than 100 action potential models (or modified versions) have been developed for different types of myocytes and species. Tissue and organ scale models, including one-dimensional (1D) cable, two-dimensional (2D) sheet, three-dimensional (3D) slab, and anatomically based ventricle and atrium models, have been developed. These mathematical and computational models have been widely used to investigate cardiac excitation-contraction coupling and arrhythmias under physiological and pathophysiological conditions.

**Figure 1:**
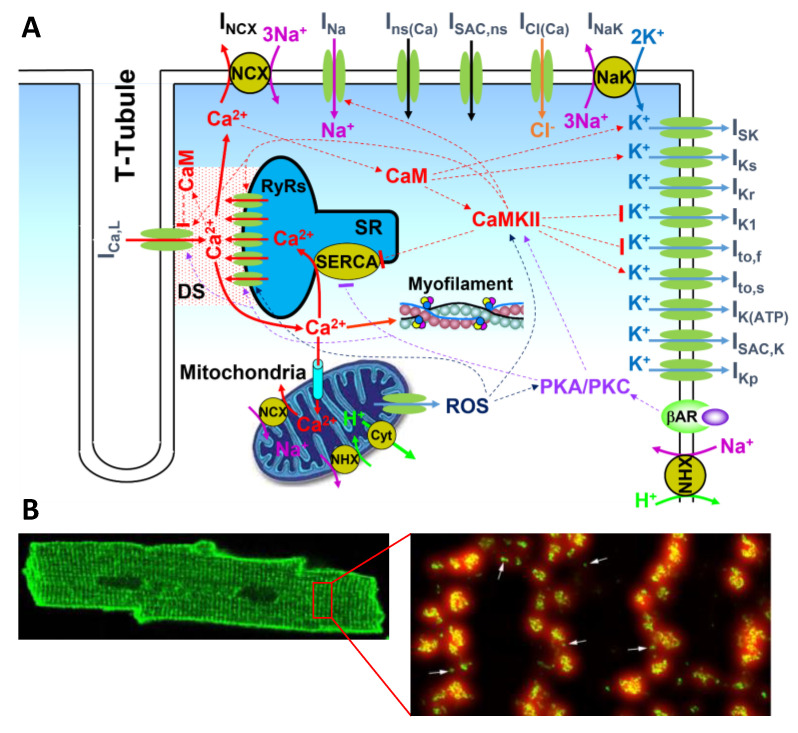
Ca cycling/signaling in cardiac myocytes. (**A**) Schematic diagram showing Ca cycling and signaling and its coupling with voltage, including the major components: (1) ionic currents and their regulation by Ca/calmodulin (CaM) and Ca/calmodulin-dependent protein kinase II (CaMKII); (2) SR Ca release and uptake and their regulation by CaMKII and protein kinase A and C (PKA and PKC); (3) mitochondrial Ca cycling; (4) Ca and myofilament interaction; and (5) Ca-dependent signaling pathways. DS stands for dyadic space and ROS for reactive oxygen species. For a detailed explanation of each component in the diagram, see Ref. [22]. (**B**) RyR clusters measured from a ventricular myocyte, adapted from Ref. [9].

Modeling of the voltage in the heart is relatively well executed, mainly following the Hodgkin–Huxley (HH) modeling approach [6]. The governing equation for the transmembrane potential (V) of a myocyte is simply described by the following differential equation: $\frac{dV}{dt}=-I_{ion}/C_{m}$, in which $I_{ion}$ is the total ionic current density and $C_{m}$ is the cell membrane capacitance. In cardiac myocytes, there are many types of ionic currents (Figure 1A) which are modeled either using the HH formulation or Markovian approaches. In the HH formulism, the ionic current density is described as $I_{s}=G_{s}x^{m}y^{n}z^{k}\left(V-E_{s}\right)$, in which $G_{s}$ is the maximum conductance, and $E_{s}$ is the reversal potential. *x*, *y*, and *z* are the gating variables described by differential equations with properties (steady states and time constants) from experimental measurements of whole-cell voltage clamp recordings [6]. In the Markovian approaches, there are two ways of modeling. In the first way, single ion channel openings and closings are simulated using stochastic Markovian transitions, and the total ionic current of an assembly of ion channels is described as $I_{s}=g_{s}N_{o}\left(V-E_{s}\right)$, in which $g_{s}$ is the single-channel conductance and $N_{o}$ is the number of open channels at a given time. In the second way, differential equations are used to describe the probabilities of states in the Markovian scheme, and the ionic current of an assembly of ion channels is described as $I_{s}=G_{s}P_{o}\left(V-E_{s}\right)$, in which $P_{o}$ is the open probability of the ion channels. Note that a ventricular myocyte is a 3D entity with its dimension being roughly [7] 150 × 30 × 15 μm^3^, but in the current action potential models, voltage is considered uniform over the entire cell membrane. In other words, at any moment, the ion channels in the entire cell membrane are assumed to sense the same voltage. Moreover, in the Markovian scheme, it is assumed that the ion channels are statistically independent, and thus the whole-cell current is simply the summation of the single-channel currents.

However, Ca cycling and its dynamics are much more complex to model. Ca cycling not only is required for contraction but also plays important roles in regulating ionic currents (Figure 1A). Ca is stored in the sarcoplasmic reticulum (SR), which forms a complex network inside the cell. Ca is released from the SR into the cytoplasmic space through the opening of the ryanodine receptors (RyRs) in the SR membrane. Opening of the RyRs is activated by Ca on both the cytoplasmic and luminal sides. Under the normal condition, SR Ca release is mainly triggered by Ca entry from the L-type Ca channels (LCCs). Under diseased or Ca overload conditions, spontaneous Ca release occur more frequently. In cardiac myocytes, RyRs form clusters (Figure 1B), which combine with their associated LCC clusters to form basic units of Ca signaling, called Ca release units (CRUs). A cell contains hundreds to thousands of CRUs [8,9,10], which form a coupled network via Ca diffusion in the cytoplasmic space and SR. A rich spectrum of spatiotemporal Ca dynamics is observed in cardiac myocytes and other cell types, including Ca sparks, waves, and oscillations [11,12,13,14,15,16,17,18,19,20,21]. Due to the complex spatiotemporal Ca dynamics, it has been challenging to model Ca cycling dynamics in the cardiac system, particularly at the tissue scales. Models of different temporal and spatial scales have been developed to investigate these spatiotemporal dynamics. In this article, we review the progress of modeling of Ca cycling in cardiac systems from single RyRs to tissue scales, the pros and cons of the current models and different modeling approaches, and the challenges to be tackled in the future.

## 2. RyR Models

**Figure 2:**
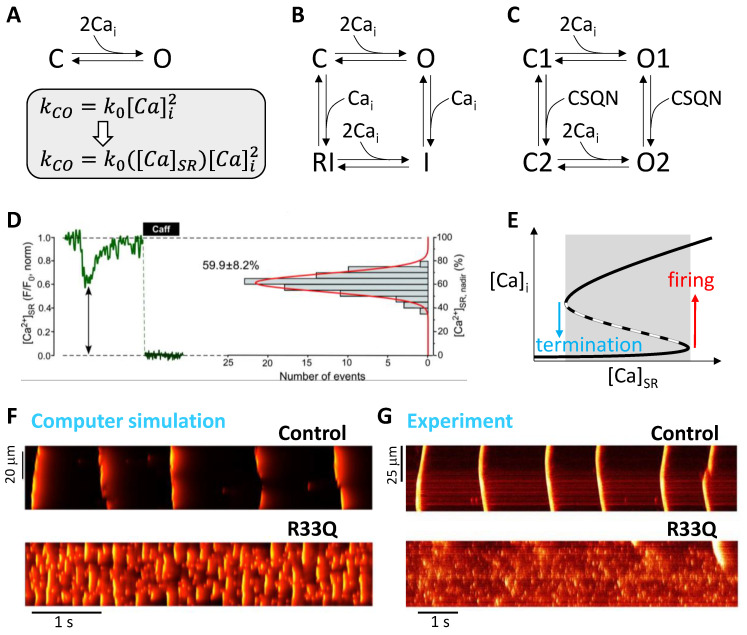
RyR models and Ca cycling dynamics. (**A**) A two-state model. The rate constant from C to O (k_CO_) as a function cytosolic Ca without or with luminal SR Ca regulation. (**B**) A four-state model with cytosolic Ca-dependent inactivation developed by Stern et al. [29,38]. (**C**) A four-state model with calsequestrin-dependent inactivation developed by Restrepo et al. [40]. (**D**) Left: SR Ca during a spark and after caffeine. Right: SR Ca nadir distribution measured during sparks. Data from Zima et al. [36]. (**E**) Bistable switch for spark firing and termination. Red arrow marks the SR Ca threshold for spark firing and cyan arrow marks the SR Ca threshold for spark termination. Gray marks the bistable region in which the high state and low state are stable and the middle one (dashed) is unstable. (**F**) Upper: Time-space plots of Ca from a computer simulation of a 3D cell with the RyR model in C. Lower: Time-space plots of Ca from a computer simulation of the same 3D cell with the RyR model without the CSQN-dependent inactivation, equivalent to the two-state model in A. Simulations were done under voltage clamped at −80 mV using the 3D cell model by Song et al. [59]. (**G**). Time-space plots of Ca from a permeablized rat ventricular myocyte of control (upper) and R33Q mutation (lower), adapted from Ref. [60].

The RyRs are regulated by Ca from both the luminal and the cytosolic sides of SR [23,24,25], and thus they are sensitive to both cytosolic and SR Ca. There are several RyR models developed and used in modeling cardiac Ca cycling. The simplest model is a two-state model consisting of a closed state and an open state (Figure 2A), used in many previous simulation studies [26,27,28]. The close-to-open transition rate is a function of cytosolic Ca. SR Ca dependence is also added to model the luminal Ca sensitivity. A key issue that has been investigated related to RyR models is how a Ca spark is terminated spontaneously [26,27,29,30,31,32,33,34], and several mechanisms have been proposed based on computer simulations. In the scenario of the two-state model, for a Ca spark to terminate, the SR Ca content needs to be depleted to a very low level (~90% depletion) to allow the RyRs to transition from the open state to the closed state. This requires a long enough time delay between the junctional SR (JSR) and the network SR (NSR). This delay is assumed to be caused by either slow SR Ca diffusion or complex NSR structures [26,27,32]. However, this assumption is not supported by experiments which show that Ca diffusion between JSR and NSR is very fast and the JSR Ca is only depleted by around 40% (Figure 2D) [35,36,37]. Stern et al. [29,38] developed a four-state model which includes inactivation and recovery states (Figure 2B), in which both activation and inactivation of the RyRs are mediated by Ca in the cytosolic space. However, there is no experimental evidence that the inactivation of RyRs is mediated by cytosolic Ca. It seems that this RyR model still requires a substantial SR Ca depletion for termination of Ca sparks [31]. Shannon and Bers [39] modified the Stern et al. model by replacing cytosolic Ca-dependent inactivation with SR Ca-dependent inactivation, which allows spark termination at a much higher SR Ca level [34]. This model also allows luminal SR Ca activation of the RyRs. Restrepo et al. [40] developed a model in which inactivation is mediated by calsequestrin in the SR (Figure 2C). Other RyR models have also been developed [29,30,41,42,43], and the effects of RyR cooperativity are investigated [26,43,44,45,46,47].

Despite the effects of RyR activation and inactivation properties on spark termination, no matter how the RyRs are activated and inactivated, there is a common dynamical mechanism of spark firing and termination [31,34], in which the firing and termination of a spark are governed by a bistable switch (Figure 2E). Namely, the cytosolic Ca concentration ([Ca]_i_) has two stable states in a range of SR Ca concentration ([Ca]_SR_) (gray region in Figure 2E). When [Ca]_i_ is at the low state, as [Ca]_SR_ reaches a threshold (red arrow), spontaneous firing occurs, and [Ca]_i_ increases to the high state. If SR depletion is fast enough to be below another threshold (cyan arrow), [Ca]_i_ quickly switches to the low state, terminating the firing and resulting in a normal Ca spark. However, if the SR depletion cannot be low enough to reach the threshold, [Ca]_i_ will stay at the high state, failing to terminate the spark promptly. The bistability is a result of the positive feedback due to Ca-induced Ca release. Whether there is RyR inactivation or not, [Ca]_SR_ is the key parameter for the termination of sparks. For the RyR models without inactivation or weak inactivation, once SR Ca is low enough, the Ca flux from the JSR to the dyadic space is so small that [Ca]_i_ in the dyadic space becomes low. The low [Ca]_i_ causes the transition from the open state to the closed state to over compete the transition from the closed state to the open state, shutting off the RyRs. For the models with RyR inactivation, particularly with SR Ca-dependent inactivation, inactivation is the primary cause for shutting off the RyRs. The difference is that the termination threshold of [Ca]_SR_ is much lower for the RyR models without inactivation.

When [Ca]_SR_ is higher than the termination threshold, the spark can still terminate due to stochastic fluctuation of RyR openings, resulting in the so-called long-lasting sparks [31,34,48,49]. This random transition is widely known as the Kramers escape process in stochastic nonlinear systems. In other words, noise can cause the system to cross the potential barrier (the unstable state, dashed line in Figure 2E) to reach the low state. Theoretical analysis by Song et al. [34] showed that the duration of the long-lasting sparks exhibits an exponential distribution, which is described by the Kramers rate theory. Exponential distributions of spark duration have been observed in cardiac myocyte experiments in which long-lasting sparks were promoted by FK506 [48]. Note that the same random transition applies to spark firings when [Ca]_SR_ is lower than the firing threshold, i.e., random fluctuations can cause transition from the low state to the high state even when [Ca]_SR_ is lower than the firing threshold, following the same Kramers transition process. For LCC-triggered sparks, [Ca]_SR_ is lower than the firing threshold, and Ca from the LCCs elevates [Ca]_i_ to cross the barrier (the unstable state) to reach the high state, firing the spark. Therefore, whether the sparks are spontaneous or triggered by L-type Ca current (I_Ca,L_), they follow the same dynamical mechanism.

Besides the spark dynamics, not surprisingly, how RyRs are activated and inactivated is also important for the cell-scale Ca dynamics, such as Ca alternans and Ca waves. For example, it is shown that RyR refractoriness is important for the formation of Ca alternans [50,51,52,53,54,55], indicating that the models with inactivation and refractoriness are more appropriate to describe the RyRs. Ca waves and oscillations are another widely observed phenomenon in the cardiac myocytes [17,56,57,58,59], and RyR refractoriness plays an important role in forming waves and oscillations. For example, removing the calsequestrin-mediated inactivation from the RyR model in Figure 2C, Ca waves become fractionated and irregular, as shown in simulations using a 3D cell model (Figure 2F). The simulation result agrees with the experimental observation that the R33Q mutation causes high-frequency but fractionated Ca waves (Figure 2G) [60], in which the R33Q mutation lacks the calsequestrin-mediated inactivation of RyRs. This implies that the two-state RyR model may not be appropriate for normal Ca cycling. Future modeling is needed to investigate how different RyR models affect the Ca alternans and Ca wave dynamics and compare them with experimental observations to further reveal the RyR models’ appropriateness.

## 3. Single CRU and CRU Network Models

**Figure 3:**
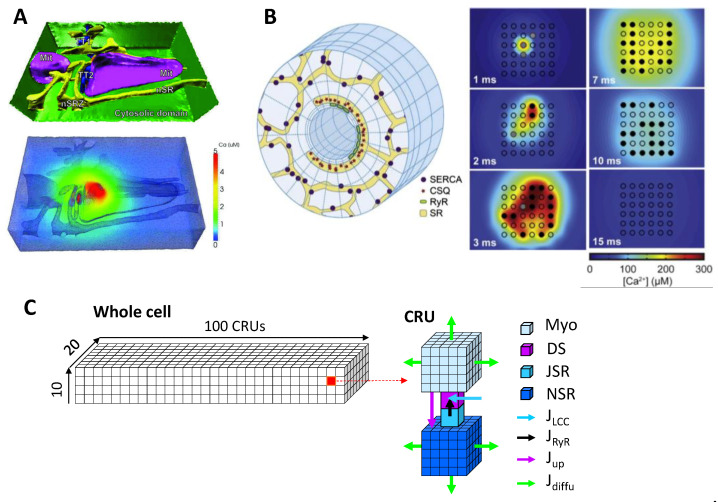
Single CRU and CRU network models. (**A**) CRU model with realistic structure, adapted with permission from Ref. [63], copyright 2012, John Wiley and Sons. Upper: Structure. Lower: A Ca snapshot during a Ca spark. (**B**) A detailed CRU model, adapted with permission from Ref. [32], copyright 2013, Elsevier. Left: Model structure. Right: Snapshots of Ca at different time points in the dyadic space. (**C**) A CRU network model to represent the whole cell [57,66]. Left: A 3D network consisting of 100 × 20 × 10 CRUs, representing a whole cell. Right: Structure of a CRU. Colored cubes mark different spaces and arrows indicate different fluxes.

Models of single CRU with different spatial resolutions have been developed. In 1992, Stern [61] put forth a theory for excitation-contraction coupling to explain the graded response of Ca release to I_Ca,L_, in which a local control (or “cluster bomb”) model is assumed. In this local control model, an LCC triggers a cluster of RyRs to open collectively, resulting in an all-or-none discretized release event. The all-or-none release events are probability events proportional to the strength of I_Ca,L_, giving rise to the experimentally observed whole-cell graded response. This cluster bomb model can be considered as the first single CRU model. In 1993, the discretized release events, called Ca sparks, were demonstrated in experiments [21]. Later, more detailed CRU models [26,29,38,62] that include stochastic RyR openings and different compartments were developed to investigate the spark dynamics, especially the mechanism of termination of a spark as described earlier. Higher spatial resolution and more realistic CRU models were also developed, including realistic T-tubule structure reconstructed from experiments (Figure 3A) [63], spatially distributed RyRs with Ca diffusion in the dyadic space [27,28,32,64] and complex SR network structures (Figure 3B) [27,32]. These high-resolution models provide further details on spark firing and termination properties.

**Figure 4:**
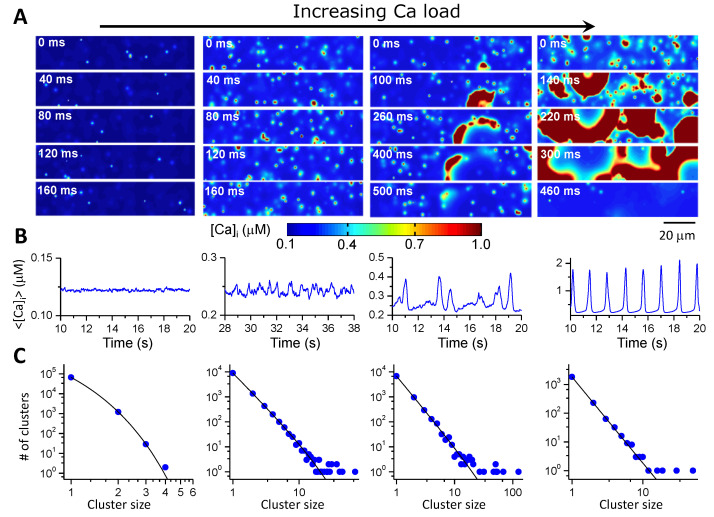
Spatiotemporal Ca cycling dynamics in a 3D CRU network model [57]. (**A**) Snapshots of Ca concentration in the cytoplasmic space for different Ca loading achieved by altering extracellular [Ca]_o_. From left to right, [Ca]_o_ = 3 mM, 9 mM, 10 mM, and 16 mM. (**B**) Averaged whole-cell Ca concentration for the corresponding loading conditions. (**C**) Number of clusters versus spark cluster size. Symbols are data from simulations and lines are reference lines. The line in the left most panel is exponential, but those in other panels are linear, indicating power-law distribution. Power-law clustering indicates that a critical phenomenon [70,71] occurs during the transition from individual sparks to waves. Adapted with permission from Ref. [57], copyright 2012, Elsevier.

To investigate the Ca cycling dynamics in a whole cell, one needs to develop a model consisting of hundreds and thousands of CRUs. It is difficult to implement the complex CRU models as in Figure 3A,B into a cell model. CRU network models [40,52,57,65,66,67,68,69] composed of simpler CRU models were developed. These models are used to investigate the spatiotemporal Ca cycling dynamics, including Ca alternans and Ca waves. As an example, Figure 3C shows a scheme of a CRU network model developed by Nivala et al. [57,58,66]. The model consists of a 3D network of CRUs. Each CRU contains four types of spaces, bulk myoplasm, dyadic space, junctional SR, and network SR. Unlike the CRU models in Figure 3A,B, the dyadic space is a single compartment in this model and thus there is no Ca diffusion, i.e., the RyRs in a CRU sense the same Ca in the dyadic space. The CRUs are coupled via Ca diffusion in the SR and cytoplasm. Figure 4 shows the spatiotemporal Ca cycling dynamics as extracellular Ca concentration increases in the same CRU network model as in Figure 3C [57], undergoing a phase transition with criticality [70,71]. These same sequences of dynamics were demonstrated in experiments of ventricular myocytes [57]. Most of the 3D whole-cell models are similar but differ somewhat in spatial resolution and structural details, e.g., some with structural complexities between those in Figure 3A–C (see Colman et al. for a detailed review [4]). Although the CRU network models lack the realistic structures of the TT and the SR networks as the real one in Figure 3A, or even as the complex one in Figure 3B, they are much more computationally convenient, and can be easily modified to simulate different subcellular structures, different cell types, and diseased conditions [68,72].

## 4. Modeling SR Ca Cycling in Single-Cell Action Potential Models

In early action potential models, such as the Noble model [73], the Beeler–Reuter model [74], and the 1991 Luo and Rudy model [75], there is no or trivial intracellular Ca cycling. Ca cycling was added in the later action potential models using different modeling approaches.

*Global RyR models*. In this type of models, one assumes that SR Ca release is uniform in the whole cell, and thus uses a single-channel RyR model to describe the whole-cell SR Ca release. We call this type of Ca release models as global RyR models. The first action potential model with intracellular Ca cycling is the DiFrancesco-Noble model for sinoatrial nodal (SAN) cells [76], in which Ca-induced Ca release based on Fabiato’s theory [77] was implemented with the SR Ca release flux (we denoted it as $J_{rel}$ in this paper) being formulated as: $J_{rel}=\alpha_{rel}{\left[Ca\right]}_{rel}\frac{{\left[Ca\right]}_{i}^{2}}{{\left[Ca\right]}_{i}^{2}+K_{m,Ca}}$. This formulation is equivalent to the two-state RyR model in Figure 2A. This Ca cycling model was modified for excitation-contraction coupling in a rabbit atrial cell model [78]. Jafri et al. [79] developed an action potential model with Ca cycling by adopting the Keizer and Levine’s RyR model [41] for SR Ca release, and Shannon and Bers [39] developed an action potential model by using a modified Stern et al. RyR model (Figure 2B), in which SR Ca-dependent activation was added and SR Ca-dependent inactivation was used. The RyR model by Shannon and Bers was also adopted by Maltsev and Lakatta for their SAN cell model [80]. In these models, $J_{rel}$ is formulated as $J_{rel}=kP_{o}\left({\left[Ca\right]}_{SR}-{\left[Ca\right]}_{i}\right)$ in which $P_{o}$ is the open probability calculated from the RyR model. Sine in this type of models, the whole cell contains a single cytosolic Ca pool and a single SR Ca pool, and thus all the RyRs in whole cell sense the same cytosolic Ca and the same SR Ca. These models are called “common-pool” models. However, as pointed out by Stern [61], the “local-control” model is more appropriate for I_Ca,L_ triggered SR Ca release exhibiting graded response. On the other hand, the common-pool model with a global RyR model allows spontaneous Ca release responsible for delayed afterdepolarizations (DADs) in ventricular cells [81] or the Ca clock in SAN cells [80].

*Phenomenological SR Ca release models*. In many of the action potential models, instead of using a RyR model, SR Ca release is modeled phenomenologically. For example, in the 1994 Luo and Rudy model [82,83], $J_{rel}$ was modeled as: $J_{rel}=G_{rel}\left({\left[Ca\right]}_{SR}-{\left[Ca\right]}_{i}\right)$ with $G_{rel}\propto f\left(\Delta Ca_{i,2}\right)\left[1-\exp\left(-\frac{t}{\tau_{on}}\right)\right]\exp\left(-\frac{t}{\tau_{off}}\right)$. $\Delta Ca_{i,2}$ is the Ca change at 2 ms after ${\dot{V}}_{max}$, and if this change is below the given threshold, $G_{rel}=0$. Since $\Delta Ca_{i,2}$ is caused by Ca entry from LCCs, thus $G_{rel}$ is I_Ca,L_-dependent. Another type of phenomenological Ca release model [84,85,86,87,88] uses the following type of formulation for $J_{rel}$: $J_{rel}=G_{rel}xy\left({\left[Ca\right]}_{SR}-{\left[Ca\right]}_{i}\right)$, in which *x* and *y* are activation and inactivation gating variables formulated in the form of HH formulism as for the membrane ionic currents. In this type of model, the activation gating *x*’s steady state ($x_{\infty }$) is a function of I_Ca,L_, and thus Ca release is I_Ca,L_-dependent. In a study by Livshitz and Rudy [89], they used a Ca release model by the following differential equation of $J_{rel}$: $\frac{dJ_{rel}}{dt}=-\frac{J_{rel,\infty }+J_{rel}}{\tau_{rel}}$, in which $J_{rel,\infty }$ is a function of I_Ca,L_ and SR Ca. This formulation was later adopted in the human model by O’Hara et al. [90]. The authors stated that they developed a two-state (closed-open) model of SR Ca release, but it is unclear how a differential equation for $J_{rel}$ can be derived from the two-state model. On the other hand, a similar equation was derived earlier by Shiferaw et al. [91] based on spark statistics (see below). Note that in these models, SR Ca release is a function of I_Ca,L_, i.e., SR Ca release is triggered by I_Ca,L_, which can effectively model the graded response of Ca release. However, if I_Ca,L_ = 0, no SR Ca release can occur in these models, indicating that spontaneous Ca release cannot be appropriately modeled. On the other hand, these models can exhibit Ca alternans [84,85,86,89]. As will be discussed below, a phenomenological spontaneous release term can be added to these models for modeling spontaneous Ca release.

*Spark statistics-based models*. Shiferaw et al. [91] took a different approach to develop an SR Ca release model based on spark dynamics and statistics, which was improved in the Mahajan et al. model [92]. The basic idea is that Ca sparks are triggered by I_Ca,L_ and the rate of spark generation is proportional to I_Ca,L_, i.e., $\frac{dN}{dt}\propto I_{Ca,L}$. On the other hand, a Ca spark has a limited lifetime, and Ca sparks disappear over time. By considering the spark birth-and-death process, they derived a differential equation for $J_{rel}$ as [92]: $\frac{dJ_{rel}}{dt}=N\left(I_{Ca,L}\right)Q\left(c_{JSR}\right)c_{JSR}-\frac{J_{rel}}{T}$, in which $N\left(I_{Ca,L}\right)$ is the number of sparks as a function of I_Ca,L_, T is a time constant related to the spark lifetime, $Q\left(c_{JSR}\right)$ is the fractional SR Ca release which can be experimentally determined [93,94]. Similar to the phenomenological models, this model cannot exhibit spontaneous Ca release but can exhibit Ca alternans. Indeed, this model is the first to reveal the role of fractional Ca release in the genesis of Ca alternans.

*Local-control models*. In 2002, an action potential model with a local-control model of Ca cycling was developed by Greenstein and Winslow [42]. In this model, thousands of CRUs with stochastic RyRs and LCCs are implemented with a common bulk cytosolic space. A simpler version was developed later [95]. Similar local-control models were developed and simulated with advanced numerical methods and mathematical approaches to speed up computation [96,97]. As expected, these models can well simulate the graded response. Moreover, it can exhibit Ca alternans [98]. Although no studies have been carried out to show spontaneous Ca release in these models, in principle, this type of model is capable of simulating spontaneous Ca release to result in Ca oscillations. A caveat of this type of model is that the Ca released from the SR enters into the common bulk cytosolic space, lacking the capability for spatiotemporal Ca cycling dynamics. Since it contains CRUs in the order of 10,000, it is computationally nontrivial and difficult to be used for tissue-scale simulations.

*Spatially extended cell models*. More recently, action potential models containing a 3D CRU network for Ca cycling have been developed to investigate Ca cycling dynamics and their coupling with voltage [40,52,57,66,67,68]. These models are truly local control models which can exhibit the whole spectrum of spatiotemporal Ca cycling dynamics (e.g., Figure 4). These models can be easily adapted for different types of cells, such as atrial myocytes [99,100,101,102,103] and SAN cells [104,105] that exhibit complex T-tubular structures [106,107], as well as subcellular structural remodeling in heart failure [72] in which T-tubule disruption occurs [108,109,110]. Other action potential models with spatially extended Ca cycling have also been developed to simulate subcellular Ca alternans and triggered activities [46,111,112,113,114,115]. A major caveat is that the spatially extended models are computationally challenging to use for tissue-scale simulations.

As mentioned above, each type of model has its advantages and pitfalls. A common issue of the non-spatial models is that they cannot properly simulate DADs. Even if some of them can simulate DADs, such as the model by Shannon and Bers [81], it cannot capture the stochastic nature of DADs [59,116,117,118] as well as the positive feedback between voltage and Ca releases that give rise to the dynamics of the triggered activity [59,116]. As shown in Figure 4, when intracellular Ca is low, such as under normal conditions, the Ca sparks are independent stochastic events (i.e., no spark-induced sparks). Thus, a statistical approach used by Shiferaw et al. [91] to derive the Ca release equation is a more rigorous approach (i.e., the differential equation is derived based on first principles) than others. Therefore, the model by Shiferaw et al. is more appropriate for the condition of low or normal Ca level. On the other hand, when Ca is very high, Ca oscillates synchronously and almost periodically; the global RyR modeling approach is more appropriate. However, none of the models (except for the spatial cell models) can correctly capture the dynamics at the intermediate range of Ca level where large random fluctuations occur due to criticality (Figure 4). This is the range where DADs and triggered activity occur.

Besides DADs, it has been shown experimentally that early afterdepolarizations (EADs), although mainly caused by reactivation of I_Ca,L_ [119], can also be caused by spontaneous Ca release [120,121,122,123]. Spatially extended 3D cell models [124,125] have been used to investigate the roles of spontaneous Ca release in the genesis of EADs, which can capture certain features of the experimental observations. Wilson et al. [126] modified a non-spatial cell model, the Shannon and Bers model [39], to exhibit spontaneous Ca oscillation and investigated spontaneous Ca release/oscillation on EAD genesis. However, in this model, the voltage depolarizations for EADs occur much earlier than the corresponding spontaneous Ca releases (the Ca oscillation and the voltage oscillation for EADs are almost in opposite phase) [126,127], which does not agree with the experimental observation that the rise of Ca occurs slightly ahead of the voltage depolarization of an EAD (experimental evidence to support that Ca release causes the EAD, not the other way around) [122,123]. Due to the spatiotemporal nature of the Ca cycling dynamics, the same problems remain for modeling spontaneous Ca release induced EADs as those for modeling DADs using the non-spatial action potential models.

## 5. Modeling Mitochondrial Ca Cycling and Energetics in Action Potential Models

Mitochondria are not only the energy store but also another Ca store for the cell (Figure 1A) and play important roles in intracellular Ca cycling and cardiac function [128,129]. Mitochondrial metabolism provides energy for ion pumps, including Na-Ca exchanger, Na-K pump, and SERCA pump. Furthermore, there are ATP-sensitive K channels and RyRs are also regulated by ATP. Besides providing energy for the ion channels and pumps, mitochondria also participate directly in intracellular Ca cycling. Cytosolic Ca enters into mitochondria via mitochondrial Ca uniporter and extrudes from mitochondria via mitochondrial Na-Ca exchanger. Ca may also get out of mitochondria via the transient opening of the mitochondrial permeability transition pore. Non-spatial (single mitochondrial compartment) models integrating mitochondrial metabolism and Ca cycling have been developed to simulate the effects of mitochondria on Ca cycling and arrhythmogenesis [130,131,132,133,134]. However, mitochondria form a network inside the cell, which can exhibit complex spatiotemporal mitochondrial depolarization dynamics, such as critical phenomenon in mitochondrial depolarization [135,136]. In our recent publications [137,138,139,140], we developed a model with a spatially distributed mitochondrial network and used it to investigate Ca alternans, EADs, and spontaneous Ca release induced DADs and triggered activity. Similar to the case without mitochondria in the model, when Ca dynamics becomes spatiotemporal, the single mitochondrial compartment model may fail to capture the Ca cycling dynamics and network models are needed.

## 6. Modeling Ca-Dependent Signaling

Ca-dependent signaling (Figure 1A) plays key roles in cardiac excitation-contraction coupling and arrhythmogenesis [22,141]. Pathways of β-adrenergic signaling, protein kinase A/C signaling, reactive oxygen species-dependent singling, and Ca/calmodulin-dependent protein kinase II signaling have been incorporated into many action potential models [87,90,138,142,143,144,145,146], which have been used to investigate their effects on action potential and Ca cycling dynamics. Most of the models are non-spatial models, and it has not been investigated how the complex spatiotemporal Ca cycling dynamics will affect the Ca-dependent signaling which then affects the Ca cycling and action potential dynamics.

## 7. Tissue-Scale Modeling for Spatiotemporal Ca and Voltage Dynamics

In most of the tissue-scale simulations, non-spatial cell models were used. As mentioned above, these models cannot properly model DADs and DAD-mediated triggered activities as well as spontaneous Ca release mediated EADs. To simulate the effects of spontaneous Ca release on arrhythmias at the tissue scale, different approaches have been used. A simple and straightforward way of modeling was used [118,147], in which stochastic DADs were phenomenologically implemented by commanding stochastic SR Ca release with statistical properties from experimental data. Chudin et al. [84] added phenomenologically a spontaneous release term to the release flux as: $J_{spon}=kP_{spon}\left({\left[Ca\right]}_{SR}-{\left[Ca\right]}_{i}\right)$, in which $P_{spon}$, described by a differential equation, is a gating variable depending on cytosolic Ca. Chen and Shiferaw [111] developed a phenomenological model by adding a spontaneous spark generation term $R_{SCR}\left(t\right)$ to the differential equation for $J_{rel}$ and used a small 1D array to calculate $R_{SCR}\left(t\right)$. This model was used in a whole-heart simulation for random DADs and triggered arrhythmias [111,148]. Colman et al. [149,150] developed a multi-scale modeling approach in which a phenomenological spontaneous release function was derived based on the detailed 3D single-cell simulations, and used it to simulate DAD-mediated arrhythmias in tissue models. More recently, a phenomenological model for Ca wave induced APD alternans in atrial cells was developed and used in atrial tissue for alternans induced arrhythmias [99,151]. Walker et al. [152] simulated a 100-cell cable with a detailed 3D cell model and developed a spatial-average filtering method for estimating the probability of extreme stochastic events from a limited set of spontaneous Ca^2+^ release profiles to predict DAD events in tissue.

Although these simulation studies have provided useful information and mechanistic insights into DAD-mediated arrhythmias, they have their pitfalls. One major issue is the effects of the feedback between Ca and voltage when complex spatiotemporal dynamics occurs. This feedback is lacking in the current multi-scale modeling approaches. Moreover, tissue simulations with detailed 3D cell models are needed to validate the results of the phenomenological models, however, this is computationally non-trivial for large tissue sizes.

**Figure 5:**
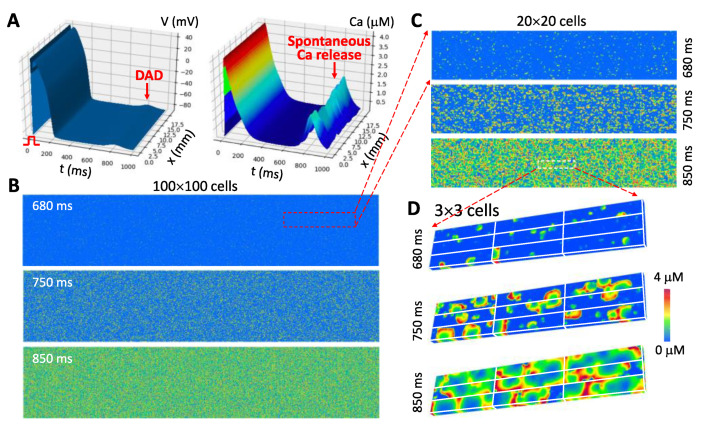
Computer simulation of a 100 × 100-cell 2D tissue using a 3D spatial cell model showing spontaneous Ca release and DAD following a paced beat. (**A**). Voltage (left) and whole-cell Ca transient (right) from a line of 100 cells in the tissue. The action potential was elicited by a stimulus applied at t = 0. (**B**). Ca snapshots (recorded on the surfaces of the 3D cells) in the 100 × 100-cell tissue taken from three time points as marked. (**C**). A zoom-in view of Ca snapshots from a small area (20 × 20 cells) for the three time points. (**D**). A zoom-in view of Ca snapshots from an even smaller area (3 × 3 cells) showing stochastic Ca wave dynamics inside the cells. Plotted is a 3D surface view of Ca on the surface of the 9 cells. The 3D cell model consist of 100 × 20 × 10 CRUs and the cell model is the same as in Song et al. [59]. The tissue model contains 2 × 10^10^ RyRs (four-state model in Figure 2C) and 2 × 10^9^ LCCs, both are simulated with stochastic Markovian transitions. The time step for the simulation is Δt=0.01.

We recently developed a parallel computational method using multiple graphics processing unit (GPU) cards, which allows us to simulate large tissue sizes with a 3D cell model. Figure 5 shows one of our simulations (unpublished data) in which a DAD occurs after a pacing beat in a 100 × 100-cell tissue using a detailed 3D cell model that is the same as in Song et al. [59]. Figure 5A shows the voltages and whole-cell Ca transients from a line of 100 cells in the tissue. Figure 5B shows Ca snapshots from the surface of the entire 100 × 100-cell tissue at three different time points as marked. Figure 5C,D show zoom-in views of Ca snapshots from a 20 × 20-cell area (Figure 5C) and a 3 × 3-cell area (Figure 5D). As shown in Figure 5D, during the DAD, the Ca waves occur in the cells, and the waves are randomly different from cell to cell.

In the simulations shown in Figure 5, the 3D cell model contains 100 × 20 × 10 CRUs, and thus the tissue contains a total of 2 × 10^8^ CRUs. Since each CRU has 100 RyRs and 10 LCCs, thus the tissue contains 2 × 10^10^ RyRs and 2 × 10^9^ LCCs. The RyRs are described by a four-state Markovian model (Figure 2C) and the LCCs are described by a seven-state Markovian model. The simulation was carried out on 10 Nvidia GTX 3090 cards using ~41.5 GiB GPU memory in total. The time step for the simulation was 0.01 ms. It costs ~3.5 computational hours to compute 1 s of the model time. Of note, our method is computationally scalable, meaning that we can perform larger tissue-size simulations with more available GPU cards within a reasonable computational run time.

## 8. Challenges for Future Modeling

Despite the progress in modeling Ca cycling at different scales in the heart, there are still plenty of challenges. A general problem is how to develop low-dimensional models or multi-scale modeling approaches to link the dynamics from the protein scale to the whole heart. Several gaps need to be filled:

(1) The first gap is to simulate sparks from a high spatial resolution CRU model to a non-spatial CRU model. As shown in the high spatial resolution models [27,28,32] (see also Figure 3B), Ca spark dynamics are spatiotemporal and depend on the spatial distribution of the RyRs in the dyadic space. Moreover, Maltsev et al. [64] showed that the occurrence of Ca sparks harnesses the Ising phase transition in a 2D array of RyRs. On the other hand, the majority of the single CRU and CRU network models ignore these spatiotemporal properties by using a RyR cluster without spatial placement of RyRs. How to correctly capture the properties of the phase transition and large fluctuations of the spatiotemporal system using a lower-resolution or non-spatial model needs to be investigated.
(2) The second gap is from CRUs (sparks) to a low-dimensional whole-cell model (Ca waves and whole-cell Ca transient). So far, using the 3D cell model for large tissue and whole-heart simulations is computationally nontrivial. Low-dimensional representations of the cell are preferred. As shown extensively by experiments and simulations (e.g., Figure 4), there is a hierarchy of Ca dynamics: quarks, sparks, spark clusters, mini-waves, persistent waves, and whole-call oscillations. How to develop a low-dimensional model to embrace these dynamics is a nontrivial challenge. As mentioned above, when Ca is low or normal, the differential equation describing the SR Ca release by Shiferaw et al. is a correct approach. At very high Ca where SR Ca release is synchronous, the global RyR model is appropriate. However, neither of the two modeling approaches can capture the Ca dynamics in the intermediate Ca range. As shown in Figure 4 (see Nivala et al. [57]), a second-order phase transition occurs for the transition from independent individual spark dynamics to the whole-cell oscillations, i.e., a critical phenomenon exists. How to model the dynamics at this phase transition using a low-dimensional representation is unknown since when criticality occurs, the dynamics is intrinsically high-dimensional. Another challenge is how to model complex 3D T-tubular structures in low-dimensional models. For example, T-tubules are disrupted in failing ventricular myocytes [108,109,110], which can cause nontrivial changes in both Ca cycling and action potential dynamics [68,72]. Correctly modeling these effects is essential for tissue-scale modeling of heart failure. Furthermore, when Ca cycling dynamics becomes spatiotemporal, their effects on Ca-dependent ionic currents and signaling, such as I_Ca,L_ and I_NCX_ as well as Ca-activated potassium currents (Figure 1A), and thus on the action potential dynamics may be nontrivial [125]. How to model these effects using a low-dimensional model is another issue needed to be concerned.
(3) The third gap is from a single cell to a syncytium (tissue and whole heart). For tissue or whole-heart simulations, cardiac tissue is treated as a syncytium with discretization in computer simulations being typically from Δ*x* = Δ*y* = Δ*z* = 0.1 to 0.5 mm [3,153,154,155]. The dimension of a typical myocyte is 0.15 × 0.03 × 0.015 mm^3^, which indicates that one “computational cell” is equivalent to 15 to 1500 real cells. Under normal conditions in which conduction is fast and Ca is mainly determined by I_Ca,L_ (so that Ca is synchronized by I_Ca,L_ in different cells), this type of discretization is appropriate. However, under diseased conditions, such as ischemia and heart failure, cells are weakly coupled and the Na current is attenuated, and thus the action potential conduction in tissue is slow. For slow conduction, a discretization at the cell size is more appropriate [156,157]. Moreover, when spatiotemporal Ca dynamics, such as waves and alternans, occur in the cell, these dynamics may be dyssynchronous from cell to cell [158] (see Figure 5), and thus a resolution of the cell size may be required for investigating these dynamics. Large tissue or whole-heart simulations with a resolution at the cell size scale is computationally nontrivial.
(4) Mitochondrial Ca cycling [131,137,138,159] and Ca-myofilament interactions [160,161] play important roles in Ca cycling dynamics. How to include their effects in a low-dimensional representation of a cell is also challenging.
(5) Although stochastic Markovian models are used to simulate single ion channel dynamics, they are still phenomenological models describing the transitions of an ion channel among different hypothetical states. Molecular dynamics simulations of single ion channels at the atomic scale are more accurate representations, which can provide parameter information for transition rates of Markovian models used in cell and tissue simulations, linking the molecular scale effects of gene mutations or drugs to cellular and tissue scale behaviors [162,163]. However, molecular dynamics simulation is computationally tedious [164], and it is still a major challenge to use it for the development and validation of Markovian models.

The central issue among these challenges is dimension reduction. Coarse graining and mean-field theories are typical modeling approaches to reduce a high-dimensional representation to a low-dimensional one [165]. Approaches for dimension reduction specific to ion channel models have been developed. For example, Fox derived a Langevin equation for a population of ion channels described by stochastic Markovian transitions for the HH formulation [166]. Keener [167] showed that many Markovian models of ion channel kinetics have globally attracting stable invariant manifolds, which can substantially simplify the computation with no approximation. He showed that this applies to certain models of potassium channels, sodium channels, and RyRs. Phenomenological modeling is currently the major modeling approach that uses low-dimensional models to describe high-dimensional systems. Iterated maps are another low-dimensional representation of high-dimensional systems [53,151,168,169]. However, both phenomenological modeling and iterated maps rely on experimental data for validation and are only valid for certain conditions.

Theoretically, if one has all the details of a system, one can build up a mathematical model to describe the motion of the atoms following Newton’s laws of motion or quantum mechanics. However, it is impossible to simulate such models due to computational limitations, at least in the foreseen future. Even if one can simulate such a big system, it is still too complex to be understandable. On the other hand, a main task of science is to reduce complex systems into ones that can be grasped by the human brain and establish principles and theories which can be passed from generation to generation. Therefore, developing low-dimensional representations that can capture the dynamics or multi-scale modeling approaches that can link the dynamics at different scales is crucial for understanding complex biological systems, such as Ca cycling in the heart.

## 9. Conclusions

The heart is so far the most widely and accurately modeled organ, and it has the potential to use the models and simulations at bedside to guide treatments. Much progress has been made for the past several decades of cardiac modeling, including models from single proteins (ion channels), whole-cell Ca cycling and action potential models, and whole-heart models. Mathematical and computational methods, such as multiscale modeling approaches and advanced numerical algorithms, have been developed. These modeling efforts have greatly helped the understanding of cardiac excitation–contraction coupling and arrhythmogenesis. Despite the progress in the last several decades, there are still plentiful pitfalls in the current models, modeling approaches, and simulation methods. The challenges to overcome these pitfalls are nontrivial but highly worthwhile for the upcoming generation of modelers to pursue in their future careers.
